# “My Back Exercise App”—mHealth for Low Back Pain: Development and Usability Testing

**DOI:** 10.1007/s41666-024-00179-0

**Published:** 2024-11-29

**Authors:** Josielli Comachio, Carlos Ivan Mesa-Castrillon, Paula R. Beckenkamp, Katharine Roberts, Emma Kwan-Yee Ho, Rowena Field, Rachel K. Nelligan, Manuela L. Ferreira, Kim L. Bennell, Christopher J. Gordon, Paulo Ferreira

**Affiliations:** 1 Faculty of Medicine and Health, School of Health Sciences, The University of Sydney, Sydney, NSW 2050 Australia; 2 Faculty of Medicine and Health, School of Rural Health, The University of Sydney, Orange, NSW 2800 Australia; 3 Department of Physiotherapy, Centre for Health, Exercise and Sports Medicine, The University of Melbourne, Melbourne, VIC Australia; 4 The George Institute for Global Health, University of New South Wales, Sydney, NSW 2000 Australia; 5 Faculty of Medicine, Health and Human Sciences, Macquarie University, Sydney, NSW 2113 Australia; 6 CIRUS, Centre for Sleep and Chronobiology, Woolcock Institute of Medical Research, Sydney, NSW 2113 Australia

**Keywords:** Low back pain, App-based intervention, Exercise, Digital health intervention, MHealth, Mobile app, Self-management

## Abstract

Effective management of chronic low back pain (LBP) often relies on maintaining a healthy lifestyle. Mobile health (mHealth) applications provide a unique opportunity to support LBP sufferers by integrating evidence-based health education into daily routines. However, many existing mHealth apps lack scientific rigour in their development, limiting their impact. This study reports on developing and usability testing My Back Exercise, an mHealth app designed to support individuals managing chronic LBP through a user-centred, theory-based approach. The development followed the Double Diamond framework and Software Development Life Cycle (SDLC) model, focusing on behaviour change theory to promote healthy lifestyle habits. Usability testing involved ten users and employed the System Usability Scale (SUS) and Mobile App Rating Scale (MARS) to assess engagement, functionality, aesthetics, and information quality. Additionally, a think-aloud approach captured real-time feedback and guided iterative improvements. Results showed that the app was well-received, achieving a SUS mean score of 79 (total score of 100) and a MARS mean score of 3.7 (out of 5.0). Key intervention components included exercises, sleep management, diet, and pain education, contributing to a holistic self-management tool. This study provides a transparent roadmap for the development of evidence-based digital health tools. The My Back Exercise app offers an innovative, holistic approach to help people self-manage chronic LBP using evidence-based methods.

**Supplementary Information:**

The online version contains supplementary material available at 10.1007/s41666-024-00179-0.

## Introduction

The global prevalence of low back pain (LBP) affects over 600 million individuals, highlighting its escalating incidence and the substantial strain it imposes on public health systems and economic resources [1]. This growing concern is not only indicative of the increase in cases but also underscores the significant challenges it presents to healthcare sustainability and economic stability [2]. LBP is associated with increased healthcare utilisation [3], as it significantly impacts individuals’ ability to perform daily activities [4], leading to absenteeism from work or decreased productivity at work [5].

Defining the trajectory of LBP is complex [6, 7]. However, the significant healthcare system costs associated with LBP underscore the importance of implementing effective intervention strategies aimed at reducing unnecessary hospitalisations [8]. Preventive measures, early intervention, and comprehensive management strategies can reduce the impact of LBP on individuals and the healthcare system [9]. Integrating digital health interventions (e.g. mobile apps, telehealth, wearables, and online therapy platforms) is a promising approach for the management of LBP, not only for improving patient outcomes [10], but also to effectively support clinicians in delivering evidence-based information and facilitating informed decision-making [11].

The development of mobile health applications (mHealth apps) to promote LBP management has significantly increased over recent years representing a notable trend in digital health interventions [12–14]. Previous reviews [10, 15] have reported that digital health interventions have the potential to support people with musculoskeletal conditions by providing self-management strategies to reduce pain and by encouraging patients to engage in healthy lifestyle behaviours [14]. Maintaining user engagement, ensuring adherence to the prescribed interventions, and the presence of non-evidence-based content are common obstacles that need to be addressed to maximise the potential benefits of these digital solutions [16]. Successful LBP-specific apps therefore require simple, user-tailored mobile applications with friendly content and design [10] that combine evidence-based strategies and user-friendly interfaces.

Considering the challenges with LBP management, as well as the uptake of digital interventions, we developed the My Back Exercise app with the main goal of promoting health and supporting the self-management of chronic LBP. A user-friendly app was developed through collaborative efforts, pooling clinical expertise, research findings, user interface design, and consumer input. Therefore, this manuscript aimed to thoroughly document the development and usability assessment of the My Back Exercise mobile app.

## Methods

The development and usability process of the My Back Exercise mobile app for LBP was carried out from March 2022 until March 2024. Two fundamental frameworks were used: the Double Diamond framework [17, 18] and the Software Development Life Cycle (SDLC) model [19]. The Double Diamond framework guided the conceptualization and ideation phase, providing a structured approach to exploring ideas, understanding user needs, defining the app’s purpose, and establishing the rationale behind its development. The SDLC framework was used to guide the technical development and implementation stages of My Back Exercise. The SDLC framework ensured that the app development process adhered to software engineering principles to ensure app functionality and reliability.

**Fig. 1:**
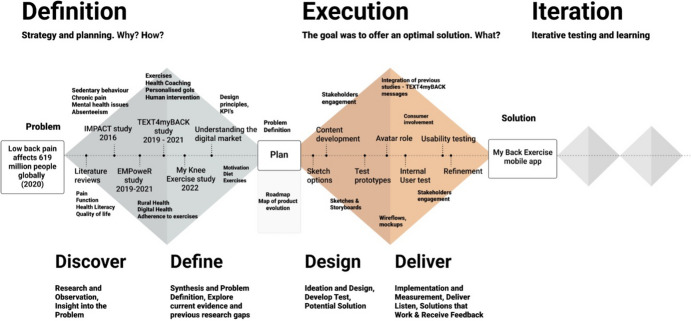
Double Diamond design process for the development of the My Back Exercise mobile app. *KPIs, key performance indicators. Design Council. (2005). The Double Diamond: a universally accepted depiction of the design process. Retrieved from https://www.designcouncil.org.uk/our-resources/the-double-diamond/

The Double Diamond framework, which guided the first phases of the app development, is presented in Fig. 1. The first diamond represents the stage of divergent thinking, where the problem space is explored widely, ideas are generated, and various possibilities are considered (Discover and Define). The second diamond represents the stage of convergent thinking, where ideas are refined, prototyped, and developed (Design and Deliver).

## Conceptualisation and Ideation

### Discover and Define

The complex nature of LBP makes behaviour change particularly challenging as it often requires adjustments to lifestyle [20], exercise routines [21], and in some cases, psychological support [22]. Educational initiatives play a crucial role in empowering individuals to manage their well-being, with recommendations to provide education and training on the characteristics and effects of pain [21, 23]. Additionally, adopting healthy behaviours such as regular exercise, better sleep habits, and improved nutrition can significantly reduce discomfort and help prevent the recurrence of related health issues [24, 25].

Our previous studies [26] [27] have focused on innovative approaches to manage LBP and knee osteoarthritis (OA), highlighted the significance of tailored interventions and the role of technology in improving health outcomes, and collectively provided compelling evidence supporting the development of the My Back Exercise app. These studies highlight the benefits of tailored exercise programs and digital health coaching, underlining the importance of individualised, accessible care solutions for chronic condition management. The “IMPACT Study” [26], with 90 participants, utilised personalised telephone health coaching, and post-treatment discharge, to enhance physical activity levels, achieving high participant satisfaction (mean = 8.7/10). Although the intervention showed a 38% reduction in the care-seeking rate among the intervention group compared to those receiving standard care, it failed to reach statistical significance (incidence rate ratio (IRR), 0.62; 95% CI 0.32 to 1.18; *p* = 0.14), underscoring the need for sustained adherence and personalised management strategies for LBP.

Conversely, the “EMPoweR Study” [27], conducted with 156 individuals in rural Australia, employed a technology-driven approach, leveraging digital platforms and remote monitoring to offer accessible, customised care for managing LBP and knee OA. Participants engaged in challenging activities, facilitated by tailored exercise prescriptions during health coaching sessions, providing valuable insights into common activities requiring improvement and the effectiveness of resistance exercises. Results of this trial showed that a 3-month real-time physiotherapist-delivered eHealth intervention, consisting of a physical activity plan and an individualised resistance training program, provided benefits by improving function in people with chronic non-specific LBP or knee OA when compared to usual care (e.g. pain medications and general practitioner care). However, certain limitations were acknowledged, including participant dropouts for 6 months follow-up due to the 2020 Australian summer bushfire season, which significantly impacted rural and remote communities in New South Wales, Australia. Additionally, the 2020/2021 COVID-19 pandemic posed challenges, complicating participants’ engagement in telehealth sessions.

The TEXT4myBACK [28] randomised trial uses text messages to support online self-management for people with non-persistent LBP [29, 30]. This study evaluates the effectiveness and cost-effectiveness of a self-management text message intervention (including pain coping strategies, advice on physical activity, sleep and health care use) compared to a control group (LBP and healthy diet information package) for improving function in people with acute LBP over a 12-week period.

These trials have highlighted the importance of holistic approaches that combine evidence-based information and digital interventions with personalised guidance, acknowledged the complexities of individual needs, and considered the potential impact of external factors (e.g. natural disasters, pandemics) on participant engagement. Challenges in maintaining consistent participation highlighted the transition to scalable digital health solutions, offering a way to overcome traditional barriers such as the necessity for in-person interactions.

The impetus for the My Back Exercise app also came from the success observed in patients with knee osteoarthritis (OA) using self-directed digital resources for OA management, as evidenced by the My Knee Exercise website [31]. This website provided OA education and a 6-month self-directed knee strengthening exercise regimen supported by automated text messages to encourage exercise adherence. The intervention was found to improve pain and physical function outcomes in people with knee OA at 6 months when compared to a control intervention consisting of online exercise/physical activity information alone. In a nested qualitative study, participants were found to have mostly positive experiences with and attitudes towards the use of a self-directed digital exercise intervention [32]. The website serves as a digital platform that enhances patient access to approved exercise programs for OA management and supports healthcare professionals in providing exercise-based treatments.

#### Understanding the Digital Market

A search of the Android Google Play Store and iOS App Store was conducted in March 2022. This snapshot search was limited to the search term “back pain” and focused on generating a broad understanding of the back pain app market. Apps were identified (regardless of subscription fees) if they specifically targeted LBP. Search results were then downloaded and reviewed by a single reviewer (JC), who identified and categorised available features. The identified features were then incorporated into an Excel spreadsheet to assess features such as technical aspects, target population, and theoretical background. There were 69 mHealth apps identified that provided exercises and education for people with LBP. Only 18 of these apps provided an intervention program based on principles of gradual intervention progression and are available across countries such as Denmark, Australia, the United States of America, and Sweden. Similar search strategies have been employed in previous studies to systematically identify and categorise mHealth apps [12, 13, 33], ensuring a thorough evaluation of features related to technical aspects, target populations, and theoretical frameworks in various health conditions, including LBP.

### Design and Deliver

The integration of digital health strategies not only enhances patient engagement but also offers a new tool for the healthcare sector, allowing patients to participate in their well-being actively [13]. As we continued to gather insights from our previous trials and the literature on digital interventions for chronic musculoskeletal conditions, it was evident that the advancements in technology and the literature for chronic musculoskeletal conditions emphasise the significance of empowering patients through digital tools to manage chronic conditions more effectively [14, 31].

The concepts and rationale supporting the My Back Exercise app are based on Cognitive Functional Therapy (CFT) to improve physical function [34], healthy eating habits, sleep, and pain education in people with chronic LBP. Initially, we formed a working group that included members from clinical and research fields, along with experts in app development and digital health. This group consisted of physiotherapists, app developers, and researchers in different fields such as nutrition, digital health, rural health, sleep, and musculoskeletal conditions. The strategic priorities of the working group were to assist people with LBP to self-manage their LBP, as well as to strengthen digital health foundations for LBP through evidence-based standards. The development of this app was influenced by systematic literature reviews on physical activity and exercise interventions [20, 34, 35] and a digital health approach that supported self-management of LBP [11, 13]. Additionally, we reviewed clinical practice guidelines and systematic literature searches on LBP-related factors (lifestyle, psychological, physical, and genetics) [22, 24, 36–39] and self-management [21], drew from clinical expertise, and engaged in group discussions within the working group. Subsequently, based on recommendations from the LBP literature and previous experiences with interventions targeting LBP within the working group, the overall program goal of the app was defined.

## Road Map of Product Evolution

Considering the importance of providing advice for people with LBP to remain active [40, 41] and the results from previous trials [26, 27, 31, 42] using digital health interventions to promote exercise, a consensus was reached that exercise should be the key focus and the benefits of exercise should be emphasised across modules. To describe the development of the app, we used the Software Development Life Cycle (SDLC) which refers to the process followed to design, develop, test, and implement mobile apps. Offering a structured approach to app development we divided this process into four phases.

### Phase 1: Content Development (March to December 2022)

The My Back Exercise app delivers a program of lifestyle behaviour change interventions for adults with chronic LBP. The key feature of the app is a strengthening exercise program, supported by a sleep behavioural program, educational notifications about LBP, and nutrition advice. In addition to the research team, identified stakeholders were included in the consultation meeting, including clinicians, developers, and researchers. The initial consultations with researchers and key stakeholders began in March 2022 and continued throughout the development process. Early consultations focused on establishing the scope and technical requirements for the proposed digital health intervention, before commencing written content.

The content of My Back Exercise is divided into four modules focusing on lifestyle change and improving health literacy, which are exercise, sleep tips, diet tips, and my back tips. These modules have been meticulously designed to offer essential information without overwhelming users. The objective of the modules is to ensure that users can engage with the content in a meaningful way, promoting a balanced and informed approach to improving their overall health. Furthermore, the development process for the exercise, diet, sleep, and education modules in the My Back Exercise mobile app involved a combination of text and images, and the SHeLL (Scripting in Higher-Level Language) editor [43] as our primary tool for crafting and refining the content to ensure readability.

#### Exercise Module

Research on exercise for chronic LBP is extensive, and while a comprehensive exploration of potential adverse events is lacking, the advantages of incorporating exercise for managing chronic pain are well-documented [44, 45]. Positive outcomes associated with regular exercises for individuals with chronic LBP, including improvements in functionality and pain management, have been consistently presented in the existing literature, especially when compared with pharmacological interventions [46].

The exercise program is evidence-based and aims to overcome fear and avoidance of movement, improving physical function and confidence in moving the spine normally [34, 47, 48]. The exercises were selected based on the principle of encouraging individuals to stay active, targeting major muscle groups, and focusing on enhancing overall physical function and mobility [49]. In this module, users select three activities from a list of 22 activities commonly reported as challenging due to pain (e.g. bending, gardening, and walking). After selecting the activities, users rate their level of difficulty in performing them (0—unable to perform the activity; 10—able to perform the activity at the same level as before the injury or problem). Subsequently, the app generates an exercise program (e.g. squats, core exercises, lunges, push-ups, and functional movements) based on the selected activities.

The exercise module provides a comprehensive 6-week exercise program offering video demonstrations and detailed instructions prioritising safety and comfort. In addition, to boost engagement, the exercises are presented through videos featuring real individuals showcasing exercises adapted for various needs, such as incorporating a pillow or a chair for added comfort and safety. The videos demonstrating exercises were recorded with three individuals of different age groups and incorporated over 50 pre-recorded videos, each integrating varying difficulty levels. In addition, audio and subtitles were incorporated into all exercise videos.

Acknowledging the importance of monitoring adherence, the exercise module integrates a progress-tracking feature, allowing users to systematically monitor their adherence. Moreover, users can report their weekly level of exertion for each exercise and have the option to switch twice from the suggested exercises if they find any discomfort, thereby enhancing the program’s adaptability to individualised preferences and ensuring a comfortable user experience.

#### Sleep Module

Research indicates a consistent link between sleep disturbance and chronic pain, including lower back, neck/shoulders, and widespread bodily pain [50, 51]. Sleep disturbance has been identified as a major contributing factor to both the onset and exacerbation of LBP [51]. Recognising the intricate interplay between sleep and LBP is crucial for the development of comprehensive strategies in pain management.

My Back Exercise incorporates a sleep module, providing information and resources on sleeping habits, sleep quality, and the relationship with LBP [40, 52]. The sleep module was developed as part of a 4-week sleep education program, incorporating written materials alongside visual content. The content of the sleep module is unlocked progressively providing reminders and tips on how to improve sleep through relaxation training and stimulus control strategies [53]. The users gain access to valuable insights and practical tips aimed at improving their sleep patterns. This includes techniques, such as relaxation training accompanied by meditation audios and tips (i.e. regular sleep routine and relaxing bedtime routine).

#### Diet Module

A growing body of evidence indicates that inadequate nutrition, encompassing factors like malnutrition, unhealthy dietary habits, and suboptimal dietary intake, can significantly contribute to the onset, progression, and persistence of chronic pain [54]. The relationship between nutrition and chronic pain is multifaceted and encompasses various underlying mechanisms, including oxidative stress, inflammation, and glucose metabolism [54]. Effective pain management necessitates a holistic and interdisciplinary approach that incorporates considerations for nutrition. Recognised as an important modifiable lifestyle factor, it is suggested that nutrition plays a role in addressing chronic pain [55].

The diet module offers a comprehensive resource covering the fundamentals of nutrition and its relationship with pain, general information about food groups, ultra-processed foods, antioxidants, and the effects of sugar on health [56]. Daily lessons such as reading food product labels contribute to improving nutritional literacy and fostering informed dietary choices. A primary aim of the nutrition content is to improve the users’ comprehension of fundamental nutrition concepts that have been proven effective in managing pain [38]. For example, it is well known that weight management and control play a crucial role in chronic pain management [55, 57]. Additionally, reducing the intake of ultra-processed foods and alcohol are significant factors impacting biomarkers associated with pain [38].

#### Pain Education Module

This module aims to improve understanding and knowledge about pain. The pain education module encompasses a 6-week educational program designed to provide participants with an education program containing written information and audio tracks about non-specific LBP [28, 44, 58, 59]. LBP information is embedded using reliable external educational resources (i.e. government websites and Australian musculoskeletal pain associations) and divided by the information per week. Therefore, participants are empowered with a structured and gradual learning experience [60]. These resources aimed to contribute to a better understanding and effective self-management strategies of non-specific LBP [59].

In addition to written content within the app, users have the option to receive push notifications to their mobile devices which offer advice, motivation, and valuable insights into the nature of LBP, emphasising various aspects such as physical activity, sleep, mood, appropriate use of care, and medication [42]. These notifications are rooted in well-documented literature, drawing from recommended practices to effectively manage and alleviate the challenges associated with health conditions [31, 61].

### Phase 2: Development of the Platform (January to June 2023)

My Back Exercise aimed to develop optimal user interface features [16]. Throughout the design phase, the researchers (JC, CM, PF, PB, and EH) and software development experts held weekly online meetings and consultations. The objective was to successfully incorporate components such as clarity, familiarity with functionality, responsiveness encompassing speed, aesthetics, and interface continuity to facilitate users in understanding usage patterns. Microsoft Office, Adobe Illustrator, Adobe XD, and JavaScript, alongside integrated development environments (IDEs) such as Visual Studio Code, were utilised to facilitate the design, functionality, and development of the app. These tools played a crucial role in streamlining the collaborative process between researchers and developers, ensuring a cohesive integration of design elements and technical functionality.

**Fig. 2:**
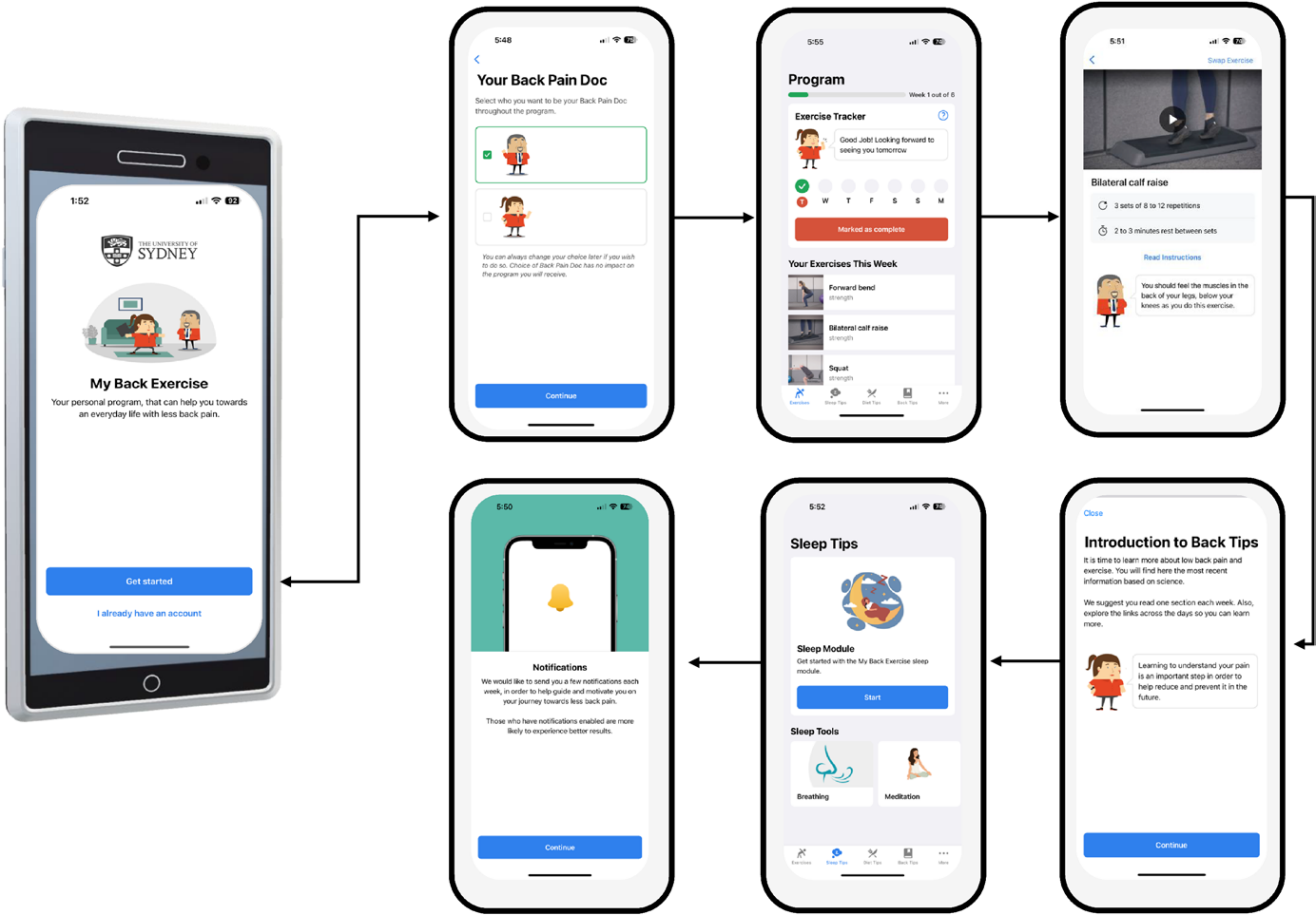
Prototype of the My Back Exercise app

The process of platform development involved several steps. Initially, a user flow diagram was drafted to visualise the application’s flow, followed by the creation of wireframes, a condensed visual representation of the app’s layout, during the initial design phase. The working group crafted a simple low-fidelity wireframe, and the software development team subsequently created a clickable mock-up simulating screen navigation. After the design phase was completed, we were able to build the My Back Exercise app during the implementation phase. The mobile application prototype was available for Android and iOS platforms in June 2023. A prototype of the My Back Exercise app is presented in Fig. 2.

#### Avatar Role

A digital representation of a therapist was developed in the form of an avatar to incorporate into the app. This avatar visually welcomes users and guides them through the app. Serving as a virtual assistant, the avatar is programmed to offer guidance and reinforce messages within the app, delivering positive motivational prompts like “Are you ready to start doing your exercises today?” and “Good job! Looking forward to seeing you tomorrow”. Furthermore, the avatar has the role of introducing some modules such as the exercise and sleep modules. The minimal interaction level introduces basic interactive elements into the avatar’s responses, incorporating simple animations and gestures to enhance engagement.

### Phase 3: Interim Period (June to December 2023)

We initiated a collaborative internal testing phase within our research team to assess the app’s performance and functionality thoroughly. Seven members (PF, CMC, JC, PB, KR, RKN, and RF) of the working group actively accessed the app, utilising its features as genuine end-users would. Post the interim period, we implemented significant improvements based on the feedback received. This included addressing issues such as spelling mistakes, refining features to enhance user experience, and promptly rectifying any technical problems or bugs identified during usage.

### Phase 4: Test and Implement User Feedback (January to March 2024)

In this phase, we implemented a user-centred design (UCD) approach to guide the development [62]. The UCD framework emphasised understanding the end-users of the app deeply, ensuring that their needs and preferences were at the forefront of decision-making. In this phase, we aimed to gather feedback from clinicians and consumers on the app using a think-aloud usability testing approach.

#### Usability Testing Using the Think-Aloud Approach

Ten experts, stakeholders, and consumer representatives specialising in digital health, LBP care, and chronic pain management were invited to participate in usability testing. These participants were from institutions including The University of Sydney, The University of Melbourne, Musculoskeletal Australia, and Sydney Local Health District, NSW, Australia. Additionally, a lived experience of LBP was included as a consumer representative. Ethics approval was obtained from the University of Sydney Human Research Ethics Committee (2023/772).

Participants were provided with detailed study information and signed informed consent forms before their involvement. Seven days before the usability testing meeting, participants received instructions via email to download and familiarise themselves with the My Back Exercise app. The app is structured as a 6-week program, but participants were asked to fast-track through the content in 1 week. This approach allowed for a more condensed yet comprehensive evaluation of the app’s usability. During the 7-day app usage period, participants also were encouraged to provide feedback via a Redcap survey before a Zoom meeting for further discussion.

As part of the remote usability testing process, online usability testing utilising the think-aloud approach also was conducted [63]. Participants were asked to verbalise their thoughts, feelings, and decision-making processes as they interacted with the app. This real-time feedback provided valuable insights into user perceptions, difficulties in navigating the app, and overall satisfaction during a Zoom meeting. The think-aloud method allowed researchers to identify key usability issues and gather qualitative data on how participants approached different tasks within the app.

#### Quantitative and Qualitative Measures

A study-specific survey was designed to collect both qualitative and quantitative feedback on the user experience.

The Mobile Application Rating Scale (MARS) [64] and the System Usability Scale (SUS) [65] were used in this phase. The MARS evaluates app quality across four dimensions: engagement, functionality, aesthetics, and information quality on a Likert-type scale. The scale consists of 23 items, and the scale often ranges from 1 to 5, with 1 indicating a lower or negative response and 5 indicating a higher or positive response [64]. The SUS scale consists of a questionnaire with a set of 10 standardised statements about the usability of a system ranging from 0 to 100. The word “app” was incorporated in the statements of the questionnaire. A score above 68 would be considered above average, indicating good usability, while anything below 68 would be considered below average, indicating poorer usability [65]. Participants were asked to rate their agreement or disagreement with these statements on a Likert scale for both questionnaires.

Materials specific to the study, including a detailed agenda for the usability meeting, and a set of predefined app usability testing tasks also were prepared. All collected data were pseudo-anonymised by assigning a participant ID and video recording securely to protect the confidentiality and privacy of participants. No monetary compensation was paid. A list of activities executed during the usability testing can be found in the supplementary file 1.

#### Data Analysis

Data collected from the MARS and the SUS were analysed using descriptive statistics to summarise participants’ ratings on app quality and usability.

Qualitative feedback from open-ended survey responses and the think-aloud session were analysed using thematic analysis. The usability agenda provided a structured approach, focusing on predefined tasks and activities related to the app’s features (e.g. navigation, content interaction, and functionality). Transcripts from think-aloud sessions were coded to identify key themes, such as ease of use, barriers to interaction, and suggestions for improvement.

### Results of the Usability Testing

The results of the usability testing conducted in Phase 4 provided valuable insights into the app’s performance and user experience. Firstly, participants were asked to provide feedback through a survey via Redcap, encompassing various aspects such as overall impressions of the app, quality and content of the app assessed by MARS, and usability assessed by SUS questionnaires after using the app for a week. These quantitative measures allowed for a structured assessment of the app’s functionality, design, and overall user satisfaction. Additionally, a more immersive approach was adopted by encouraging participants to participate in a usability testing meeting conducted in a semi-controlled environment. This session took place with a combination of a focus group approach and a think-aloud evaluation [66]. The characteristics of participants are presented in Table 1.

**Table 1:** Participants’ characteristics and overall applicability of the app

Variable	*N* = 10
Age range	
30–40	1 (10%)
41–50	6 (60%)
51–60	3 (30%)
Sex, *n* (%)	
Female	8 (80%)
Male	2 (20%)
Device	
Apple (iOS)	7 (70%)
Android	3 (30%)
**Usability questions**	**Mean (SD)**
**Enjoyability of the app: on a scale of 0 to 10, how enjoyable did you find My Back Exercise app?**	7.0 (1.4)
**Ease to use: on a scale of 0 to 10, how easy was it to use the app for the following parts of the program?**	
** Exercise**	7.8 (1.4)
** Sleep**	7.2 (1.6)
** Diet**	6.8 (1.2)
** Education**	7.0 (1.7)

Usability questions: A higher score indicates a positive response from participants.

**Fig. 3:**
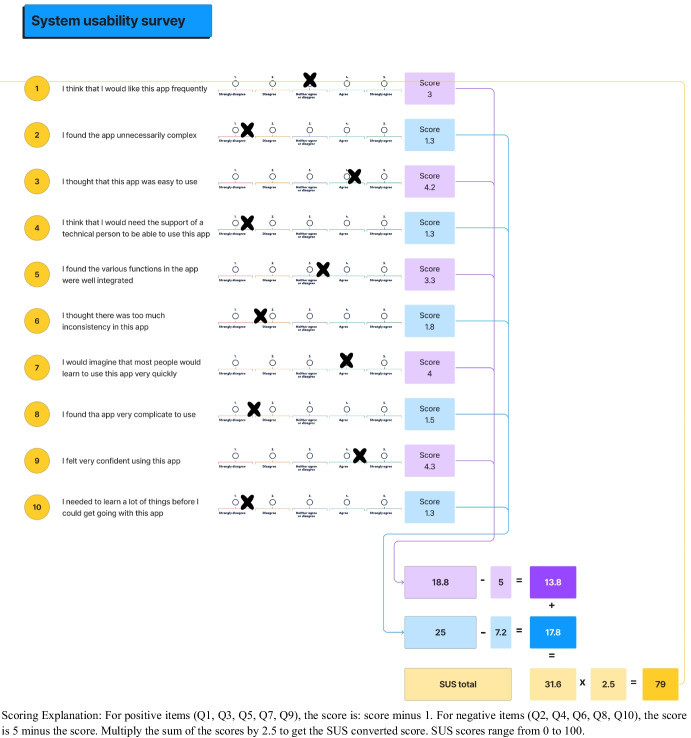
Summary of system usability survey

**Fig. 4:**
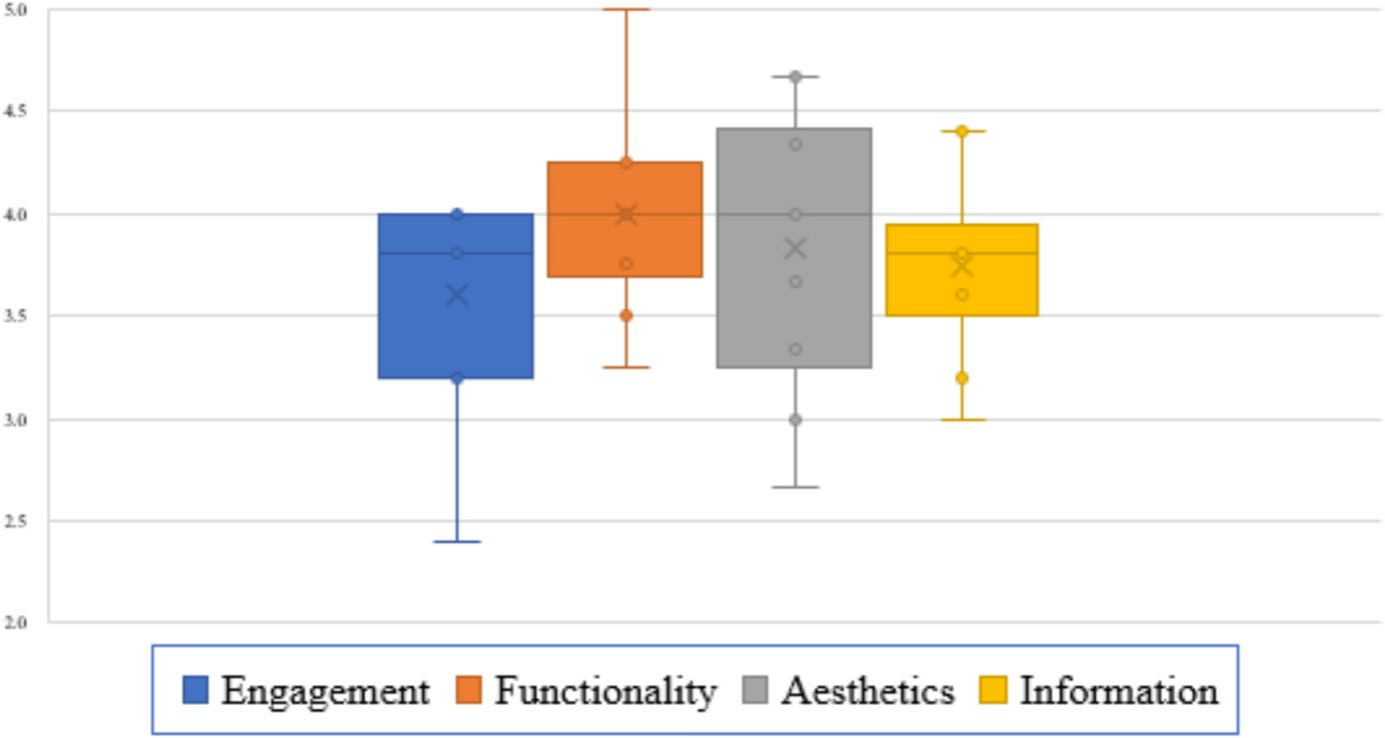
Mobile Application Rating Scale (MARS)

Of the 10 participants, 8 (80%) were female and 2 (20%) were male. Participants’ ages ranged from 30 to 60 years. The results of the SUS and MARS scores are found in Figs. 3 and 4, respectively. The obtained SUS score of 79 indicates a high level of usability and user satisfaction. This noteworthy result suggests that the app was well-received among users, reflecting a positive interaction between the design and functionality of the system.

Regarding the MARS scores, functionality achieved the highest score with a mean of 4.0 (standard deviation 0.4), followed by aesthetics with a mean of 3.8 (0.6), information with a mean of 3.7 (0.4), and engagement with a mean of 3.6 (0.5) (Fig. 4). Results presented as mean and interquartile range for each dimension: engagement, interquartile range (IQR) 3.5–4.0; functionality, IQR 3.7–4.5; aesthetics, IQR 3.8–4.5; information, IQR 3.2–3.8.

For the second round of usability testing, six (60%) out of 10 invited participants attended the meeting. This meeting included both researchers (University of Melbourne, Musculoskeletal Australia, and Sydney Local Health District, NSW) and a consumer representative experiencing LBP.

The group discussion enabled participants to share their perspectives and the research team. Simultaneously, the think-aloud evaluation encouraged participants to vocalise their thoughts as they interacted with the app, providing real-time insights into their decision-making processes and user experience. Table 2 presents the illustrative quotes from the open-ended questions and usability testing. The list of activities and quotes are presented in the supplementary files 2 and 3.

**Table 2:** Summary of feedback from open-ended survey questions and usability testing (think-aloud approach)

Questions	Summary of feedback (illustrative quotes)
What did you like about the app?	• “The user interface design is clean and logical.” • “It was simple to navigate and animated. I felt it led me on a journey that was personalised to me.” • “It is available on both IOS and Android.” • “I think the App is good conceptually.” • “It’s a tool with potential.” • “I found the exercise program section fun with the avatar component and the videos.”
What did you dislike about the app?	• “I don't like repetition. I prefer to walk. I like my comfort zone.” • “The simplicity of the program (exercises was positive) however it didn't engage me to the point that I wanted to be checking and accessing the program to update it.” • “More self-monitoring tools can be helpful (e.g. weight training log, diet log).” • “Preference would have been a recipe for a meal of the day with information built into that explaining why certain ingredients were good to eat.” • “I found the education part the least easy section to use as it contains quite a bit of text and might be better presented in video format.”

### Ethical Considerations

#### Cybersecurity

To ensure the utmost security of participant data within the cybersecurity framework, data was securely uploaded to Amazon Web Services (AWS) servers utilising 256-bit SSL encryption. Advanced security protocols were implemented to protect this data, which included the necessity for credentials with specific permissions to access the data and the requirement for multi-factor authentication to further enhance security measures. Notably, the storage of participants’ names is confined exclusively to their local devices, with this information not transmitted to the server. As a result, should a participant switch their phone or log in from a different device, they are prompted to re-enter their name. This procedure is crucial for maintaining the privacy and security of participant data.

## Discussion

This article presents the step-by-step iterative journey taken in developing a lifestyle-focused self-management mHealth app dedicated to empowering individuals living with LBP. Throughout this process, four comprehensive modules were developed (exercise, sleep, diet, and education). These modules were fine-tuned following a thorough examination of the app’s usability, focused on a user-centred design informed by consumers and researchers.

The endorsement and adoption of digital health interventions as instrumental tools for promoting lifestyle changes and self-management strategies have been robustly advocated and implemented in many countries. Despite the widespread recognition of their potential benefits such as facilitating access to health information and support, challenges persist in the development of these digital tools (e.g. most effective written content that is both engaging and informative, what strategies to enhance user engagement, and adherence to these programs). A key focus of the design of the My Back Exercise app is the evidence-based content which aligns with the recommendations of the most recent guidelines for managing LBP [41, 45].

The usability process applied in multiple phases was an important step in the development of this mHealth app. As a result, it was possible to identify issues and make necessary improvements in the early stages, leading to a more refined and user-friendly final version of the mHealth app to be further evaluated [66]. The results of the SUS score indicate that users find the app system easy to use. However, it is important to consider the slightly lower MARS scores, suggesting that the users have specific concerns or find aspects of the mHealth app less satisfactory (i.e. engagement domain). This discrepancy between the SUS and MARS scores underscored the need for a more in-depth exploration of user experiences [12, 16]. To foster a collaborative environment, we held a think-aloud session with the development team, user experience experts, and end-users to discuss key topics and concerns. This approach facilitated the sharing of insights, guiding improvements and optimizations to enhance user satisfaction across various aspects of the mHealth app. By integrating quantitative scores with qualitative user feedback, we gained a deeper understanding of user experiences and preferences.

### Strengths and Limitations

A large and diverse planning group worked continuously on developing, testing, and refining the My Back Exercise app. We applied two frameworks to guide its rigorous development, the Double diamond framework [18] and the SDLC [19]. Comprehensive pre-testing was conducted in two distinct ways: one in a semi-controlled environment via online meeting and the other through written feedback. Additionally, previous research and the team’s collective experience contributed to the app’s trustworthiness and robustness.

One potential weakness of this study is that usability testing often involves a relatively small number of participants, which may limit the representation of diverse user experiences. However, a larger sample size will be included in an upcoming randomised controlled trial to evaluate the app’s effectiveness and cost-effectiveness. We acknowledge that some participants in the usability testing were recruited based on specific criteria, such as expertise in digital health, LBP management, and health research, as well as consumer representation, which may introduce selection bias. To mitigate this, we intentionally invited participants with diverse backgrounds and experiences to contribute to the improvement process. This approach aimed to enrich the feedback and enhance the app’s adaptability for a broader audience. Our commitment to continuous improvement and a user-centric design guided this strategic decision, helping us address potential limitations and ensure that the app is more inclusive, user-friendly, and accessible to a wide range of users.

## Conclusion

My Back Exercise app was successfully developed after undergoing phases of requirement gathering, user-friendly design, implementation, and usability testing. The app is also interactive, with features that include an avatar, videos, and push notifications. Integrated content and design were developed by a multidisciplinary team, composed of consumers and experts.

My Back Exercise app will undergo testing within a community-based clinical trial to assess its effectiveness (e.g. pain intensity, physical function, sleep quality, quality of life, and overall satisfaction) in an adaptative trial design. The methodological design of the trial will aim to compare the modules (i.e. exercise, sleep, diet, and education) with education alone in a large “end-user experience” which will involve up to 370 participants. The description of the My Back Exercise app serves as a road map to developing digital interventions for people with LBP. It outlines how the app stands out in its approach and features compared to existing solutions or interventions.

It is important to understand the experiences of people experiencing LBP to improve health care and deliver person-centred care using the available technology. The clinical trial will provide the effectiveness of the mHealth app. If proven to be effective, this app will pave the path for greater adoption and deployment, which will benefit everyone looking for evidence-based self-management strategies to improve LBP.

## Supplementary Information

Below is the link to the electronic supplementary material.

Supplementary file1 (DOCX 46 KB)

## Data Availability

No datasets were generated or analysed during the current study.
