# Supplementary material for: “My Back Exercise App”—mHealth for Low Back Pain: Development and Usability Testing
Source: J Healthc Inform Res. 2024 Nov 29;9(1):67–87. doi: 10.1007/s41666-024-00179-0 (PMC11782706; doi:10.1007/s41666-024-00179-0)
Supplement: Supplementary file 1 — Supplementary file1 (DOCX 46 KB) [file 41666_2024_179_MOESM1_ESM.docx]

**SUPPLEMENTARY FILE**

**'My Back Exercise' - a mHealth app for Low Back Pain: Development and Usability Testing**

Josielli Comachio^1^ (ORCID: 0000-0002-3286-6998), Carlos Ivan Mesa-Castrillon^1,2^ (ORCID: 0000-0003-1900-5795), Paula R. Beckenkamp^1^ (ORCID: 000-0003-3986-6111), Katharine Roberts^1^ (ORCID: 0000-0003-0017-0803), Emma Kwan-Yee Ho^1^ (ORCID: 0000-0002-2479-0081), Rowena Field^1^ (ORCID: 0000-0002-1724-0321), Rachel K. Nelligan^3^ (ORCID: 0000-0002-8689-6594), Manuela L. Ferreira^4^ (ORCID: 0000-0002-3479-0683), Kim L. Bennell^3^ (ORCID: 0000-0003-4982-5639) Christopher J. Gordon^5,6^ (ORCID: 0000-0003-2698-4864), Paulo Ferreira^1^ (ORCID: 0000-0002-5861-7770)

^1^The University of Sydney, School of Health Sciences, Faculty of Medicine and Health, Sydney, New South Wales (NSW) 2050, Australia.

^2^The University of Sydney, School of Rural Health, Faculty of Medicine and Health, Orange, NSW 2800, Australia.

^3^Centre for Health, Exercise and Sports Medicine, Department of Physiotherapy, The University of Melbourne, Vic, Melbourne, Australia. 2 Medibank Private, Vic, Melbourne, Australia.

^4^The George Institute for Global Health, University of New South Wales, Sydney, NSW 2000, Australia.

^5^Faculty of Medicine, Health and Human Sciences, Macquarie University, NSW 2113, Australia.

^6^CIRUS, Centre for Sleep and Chronobiology, Woolcock Institute of Medical Research, NSW 2113, Australia.

**Table 1.** Usability Testing Activities.

| **Activity** | **Description** | **Measure** |
| --- | --- | --- |
| **Create a new account and complete onboarding** | Participants are instructed to create a new account and go through the onboarding process. | Time is taken to complete registration, ease of understanding onboarding instructions, and overall satisfaction. |
| **Quick access to commands** | Users are asked to log in and log out of the app, with a focus on identifying any issues during the process.  Prompt: Can you log in and log out of the app? Any issues? | Identification of any issues during the login/logout process. |
| **Finding and checking study email** | Participants who are stuck in the app are prompted to locate and check the study email, assessing their ability to navigate and troubleshoot within the application.  Prompt: Can you find and check the study email? | User's capability to find and check the study email when encountering difficulties. |
| **Exercise list retrieval** | Users are asked to imagine needing to check their exercise list due to forgetfulness and are tasked with finding this information within the app.  Prompt: You are not sure about the correct position to start the exercise. Can you find this information? | Ability to locate and retrieve exercise information when needed. |
| **Setting reminders for push notifications** | Participants are directed to set a reminder for push notifications, evaluating the effectiveness of reminder settings, the clarity of instructions, and user satisfaction with the reminder feature. | Effectiveness of reminder settings, clarity of instructions, and user satisfaction with the reminder feature. |
| **Exploratory task: Evaluating exercise module, sleep, diet and educational** | **Participants are presented with an open-ended question asking if they like the exercise module, allowing for subjective opinions and feedback.** | Qualitative feedback on participants' preferences and satisfaction with the exercise module. |

**Table 2**. Results based on the usability tasks and their corresponding measures

| **Activity** | **Measure** | **Results** |
| --- | --- | --- |
| **Create a new account and complete onboarding** | Time to complete registration, ease of understanding, satisfaction | Average time: 2.5 minutes. 8/10 participants found the onboarding instructions clear, overall satisfaction: 85%. |
| **Quick access to commands (Login/Logout)** | Issues encountered, time taken, satisfaction | 2/10 participants faced minor issues logging in. Average login/logout time: 30 seconds. Satisfaction: 90%. |
| **Finding and checking study email** | Ability to locate and check study email | 3/10 participants were able to find and check the study email without difficulty. |
| **Exercise list retrieval** | Ability to locate and retrieve exercise information | 6/10 participants successfully retrieved exercise information. 3/10 found navigation confusing. |
| **Setting reminders for push notifications** | Effectiveness, clarity of instructions, satisfaction | 9/10 participants set reminders successfully. 1/10 forgot to do that. |
| **Exploratory task: Evaluating exercise module, sleep, diet, and education** | Qualitative feedback on modules | Participants found the exercise module most useful (90% positive feedback), while sleep and diet modules received mixed reviews. |

**Table 3.** Quotes from users’ experience during the think-aloud session

| **Task** | **User Quote** |
| --- | --- |
| **Downloading the app** | “I thought it was very clear. I like the outlook of the app is great. I had issues downloading the app to start off with. But once I was in, it looks pretty easy to use”. *(participant 1)*  “I have nothing to add. I'd like to agree with *participant 1*, and I'll share with you. I'm not very good at doing this stuff. I call the IT services, but I found this very easy”. *(participant 2)*  “The user interface design is clean and logical.” *(participant 4)* |
| **Create a new account and complete onboarding** | “I’m not sure where to start with this... Oh, I see the button now, but it’s not very obvious, however I could not open in my computer, is this app design for iPad or computer?” *(participant 1)*  “I could not believe that I did the onboard process myself, without asking for help.” *(participant 2)*  “The onboard process was very easy, but I wonder if this possible more information on that I will find in this app, for example the modules” *(participant 3)*  “So from memory, it sounds like you needed just to create the account with your phone rather than your email, which I think is good.” *(participant 4)* |
| **Quick access to commands (Login/Logout)** | “Logging in was easy, but I can’t seem to find the logout button. Is it in the settings?” *(participant 3)*  “I login and log out but its asking my name again.” *(participant 2)*  “No problem at all.” *(participant 6)* |
| **Finding and checking study email** | “I’ve been searching for the email for a few minutes now, but I can’t seem to figure out where it is.” *(participant 2)*  “I did not find it.” *(participant 3)*  “This is so easy to find.” *(participants 4)* |
| **Exercise list retrieval** | “I got a bit stuck here.” *(participant 1)*  “Easy to find.” *(participant 5)* |
| **Setting reminders for push notifications** | “The reminders were easy to set, but it took me a while to find where to set them. I expected it to be simpler.” *(participant 1)* |
| **Exploratory task: Evaluating exercise module** | “I really like the exercise module, it’s clear and easy to follow, but the sleep section doesn’t seem as helpful.” *(participant 3)*  I answered the questions about myself, what pain, those sorts of things, got the exercises and went, Oh, yeah, the exercises look simple enough. That's great. *(participant 5)* |
| **Evaluating exercise, sleep module, diet, and education** | “I found the exercise program section fun with the avatar component and the videos.” *(participant 2)*  “You've got your exercise program, you're like, Great!... where there wasn't anything really prompting me to explore the other parts of the app.” *(participant 3)*  “I agree with participant 3.” *(participant 1)*  “... I have to go into those modules, which I did anyway, because I'm a very curious person, but I thought maybe they were there just for information. It would be good if it was clear why they're there and how they're an essential part of this program.” *(participant 6)*  “I can access everything very easy, watch the videos, it’s very helpful” (*participant 5)*  “Sleep module, the language could be clearer” *(participant 3)*  “Diet module could be harder to engage, because I don’t like diet, people don’t like to talk about this, this probably needs more engagement, because it’s hard to talk about diet” *(participant 4)*  “The diet module I feel so many words.” *(participant 5)*  “I feel the diet module is very empower, even I knew some content.” *(participant 1)* |
| **Avatar feedback** | “I loved the avatar; I don’t feel alone.” *(participant 2)*  *“*It’s a tool with potential… This is a good way to keep people engaged” *(participant 4)*  “I found the exercise program section fun with the avatar component and the videos.” *(participant 6)* |

**Table 4.** Mobile App Rating Scale (MARS).

| **Participant ID** | **Engagement** | **Functionality** | **Aesthetics** | **Information** |
| --- | --- | --- | --- | --- |
| 1 | 3.8 | 4.2 | 4.6 | 4.4 |
| 2 | 4.0 | 3.5 | 4.0 | 3.8 |
| 3 | 3.8 | 4.0 | 3.6 | 3.8 |
| 4 | 4.0 | 5.0 | 4.0 | 4.4 |
| 5 | 3.2 | 4.0 | 4.0 | 3.8 |
| 6 | 3.8 | 4.0 | 3.0 | 3.8 |
| 7 | 2.4 | 3.2 | 3.3 | 3.0 |
| 8 | 3.8 | 4.0 | 4.6 | 3.6 |
| 9 | 3.2 | 4.2 | 4.3 | 3.6 |
| 10 | 4.0 | 3.7 | 2.6 | 3.2 |
| **Total score (mean and SD)** | 3.6 (0.5) | 4.0 (0.4) | 3.8 (0.6) | 3.7(0.4) |

The rating scale assesses app quality using four dimensions. All items are rated on a 5-point scale from “1. Inadequate” to “5. Excellent”.

**Table 5.** The System Usability Scale (SUS) raw and converted scores

| ID | I think that I would like to use this app frequently  (Raw/Conv) | I found the app unnecessarily complex to use  (Raw/Conv) | I thought the app was easy to use  (Raw/Conv) | I think that I would need the support of a technical person  (Raw/Conv) | I found the various functions in the app were well-integrated  (Raw/Conv) | I thought there was too much inconsistency in the app  (Raw/Conv) | I would imagine that most people would learn to use this app very quickly  (Raw/Conv) | I found the app very complicated to use  (Raw/Conv) | I felt very confident using the app  (Raw/Conv) | I needed to learn a lot of things before I could get going with this app  (Raw/Conv) | Total score | SUS score |
| --- | --- | --- | --- | --- | --- | --- | --- | --- | --- | --- | --- | --- |
| 1 | 2 / 1 | 1 / 4 | 4 / 3 | 2 / 3 | 4 / 3 | 1 / 4 | 4 / 3 | 2 / 3 | 4 / 3 | 1 / 4 | 31 | 77.5 |
| 2 | 2 / 1 | 2 / 3 | 5 / 4 | 1 / 4 | 4 / 3 | 1 / 4 | 4 / 3 | 1 / 4 | 5 / 4 | 1 / 4 | 34 | 85 |
| 3 | 3 / 2 | 2 / 3 | 5 / 4 | 1 / 4 | 4 / 3 | 2 / 3 | 4 / 3 | 1 / 4 | 5 / 4 | 1 / 4 | 34 | 85 |
| 4 | 4 / 3 | 1 / 4 | 4 / 3 | 1 / 4 | 4 / 3 | 2 / 3 | 5 / 4 | 2 / 3 | 5 / 4 | 1 / 4 | 34 | 85 |
| 5 | 4 / 3 | 1 / 4 | 5 / 4 | 1 / 4 | 5 / 4 | 1 / 4 | 4 / 3 | 2 / 3 | 4 / 3 | 4 / 1 | 33 | 82.5 |
| 6 | 4 / 3 | 2 / 3 | 3 / 2 | 1 / 4 | 2 / 1 | 3 / 2 | 3 / 2 | 2 / 3 | 3 / 2 | 1 / 4 | 26 | 65 |
| 7 | 3 / 2 | 1 / 4 | 4 / 3 | 1 / 4 | 4 / 3 | 2 / 3 | 4 / 3 | 1 / 4 | 5 / 4 | 1 / 4 | 34 | 85 |
| 8 | 3 / 2 | 1 / 4 | 4 / 3 | 1 / 4 | 2 / 1 | 2 / 3 | 4 / 3 | 1 / 4 | 4 / 3 | 1 / 4 | 31 | 77.5 |
| 9 | 3 / 2 | 1 / 4 | 4 / 3 | 1 / 4 | 2 / 1 | 2 / 3 | 4 / 3 | 1 / 4 | 4 / 3 | 1 / 4 | 31 | 77.5 |
| 10 | 3 / 2 | 1 / 4 | 4 / 3 | 2 / 3 | 2 / 1 | 2 / 3 | 4 / 3 | 2 / 3 | 4 / 3 | 1 / 4 | 29 | 72.5 |
| Mean | 3.1 / 2.1 | 1.3 / 3.7 | 4.2 / 3.2 | 1.3 / 3.7 | 3.3 / 2.3 | 1.8 / 3.2 | 4 / 3 | 1.5 / 3.5 | 4.3 / 3.3 | 1.3 / 3.7 | 31.7 | 79.2 |

**Conv:** converted. **Scoring Explanation:** For **positive items** (Q1, Q3, Q5, Q7, Q9), the score is: **score minus 1**. For **negative items** (Q2, Q4, Q6, Q8, Q10), the score is: **5 minus the score**. Multiply the sum of the scores by 2.5 to get the **SUS converted score**. SUS scores range from **0 to 100**.
